# CLEC3B protects H9c2 cardiomyocytes from apoptosis caused by hypoxia via the PI3K/Akt pathway

**DOI:** 10.1590/1414-431X20209693

**Published:** 2020-07-17

**Authors:** Fenghua Lv, Zhuo Wang, Yanli Huang, Aoyang Si, Yulei Chen

**Affiliations:** Department of Cardiology, The First Affiliated Hospital of Xinxiang Medical University, Xinxiang, Henan, China

**Keywords:** Ischemic heart disease, H9c2, CLEC3B, PI3K/Akt pathway, Cell apoptosis

## Abstract

Ischemic heart disease (IHD) is one of the leading causes of death worldwide. C-type lectin domain family 3 member B (CLEC3B) is a C-type lectin superfamily member and is reported to promote tissue remodeling. The serum levels of CLEC3B are downregulated in patients with cardiovascular disease. However, the molecular mechanisms of CLEC3B in IHD is not well-characterized. Therefore, we overexpressed CLEC3B and silenced CLEC3B in H9c2 rat cardiomyocytes for the first time. We then constructed a model of IHD *in vitro* through culturing H9c2 cardiomyocytes in serum-free medium under oxygen-deficit conditions. Then, Cell Counting Kit-8 (CCK-8), flow cytometry, qRT-PCR, and western blot assays were performed to investigate cell viability, apoptosis, and expression levels of CLEC3B, phosphatidylinositol 3-kinase (PI3K), phosphorylated PI3K (p-PI3K), protein kinase B (Akt), phosphorylated Akt (p-Akt), and cleaved-caspase 3. We observed that the mRNA expression of CLEC3B was decreased in hypoxic H9c2 cardiomyocytes (P<0.05). Overexpression of CLEC3B increased cell viability (P<0.01), inhibited cell apoptosis (P<0.05), upregulated the levels of p-PI3K/PI3K and p-Akt/Akt (P<0.01 or P<0.05), and downregulated expression of cleaved-caspase 3 (P<0.001) in hypoxic H9c2 cardiomyocytes while silencing of CLEC3B caused the opposite results. Inhibition of the PI3K/Akt pathway reversed the protective effect of CLEC3B on hypoxic H9c2 cardiomyocytes. Our study demonstrated that CLEC3B alleviated the injury of hypoxic H9c2 cardiomyocytes via the PI3K/Akt pathway.

## Introduction

Ischemic heart disease (IHD) refers to a disease caused by a decrease in myocardial blood supply, which leads to damage and changes in myocardial function and structure. IHD is one of the leading causes of death worldwide and causes a serious threat to human health (1,2). Although the diagnosis and treatment of IHD have been greatly developed, the disease remains a major life-threatening challenge (3–5). Thus, there is an urgent need to study the mechanisms of IHD and find its potential therapeutic targets.

It is well-known that the PI3K/Akt signaling pathway regulates numerous cellular activities, including cell proliferation, apoptosis, and metabolism (6,7). An increasing number of studies have shown that the alleviation of hypoxic injury or hypoxia/reoxygenation-induced injury of cells is accompanied by activation of the PI3K/Akt signaling pathway (8,9).

C-type lectin domain family 3 member B (CLEC3B) is one of the C-type lectin superfamily members and encodes tetranectin protein (10,11). It locates in plasma, extracellular matrix, and exosomes (12). Tetranectin is a plasminogen-binding protein that promotes the activation of plasminogen, and it has been considered to affect tissue remodeling (13). CLEC3B has been reported to regulate several diseases. Overexpression of CLEC3B can promote fracture healing (13), inhibit neuronal apoptosis in Parkinson's disease (14), and inhibit the proliferation of clear cell renal cell carcinoma (11). Furthermore, some studies revealed that serum levels of CLEC3B were downregulated in patients with cardiovascular disease (15,16). However, the potential effects of CLEC3B on IHD progress are unclear.

In this study, we investigated the effects of CLEC3B on cell viability and apoptosis of H9c2 cardiomyocytes under hypoxic conditions *in vitro*. We also explored the potential of CLEC3B signaling pathway in the hypoxia model. This study explored the regulation of CLEC3B in IHD through *in vitro* experiments and provided a new idea for the treatment of IHD.

## Material and Methods

### Materials

PI3K/Akt pathway inhibitor LY294002 was purchased from Sigma Aldrich (USA). The Annexin V-APC/PI apoptosis kit was obtained from Abnova (China). RIPA lysis buffer was purchased from Beyotime (China) and the BCA protein assay kit was purchased from Thermo Scientific (USA). Cell Counting Kit-8 (CCK-8) was obtained from Dojindo (Japan). Antibodies against PI3K, p-PI3K, Akt, p-Akt, cleaved-caspase 3, and β-actin were obtained from Abcam (UK). Efficient chemiluminescence (ECL) kit was obtained from Millipore (USA).

### Cell culture and treatment of hypoxia

Rat H9c2 (2-1) cardiomyocytes were purchased from the Cell Bank of Type Culture Collection of Chinese Academy of Sciences (China). H9c2 cardiomyocytes were cultured in Dulbecco's modified eagle medium (DMEM) with 10% fetal bovine serum (FBS) (Hyclone, USA) and 1% penicillin-streptomycin (Sigma-Aldrich, USA). H9c2 cardiomyocytes were seeded into a 25-cm^2^ cell culture bottle (Corning, USA) at a density of 1×10^4^ cells/cm^2^ and maintained at 37°C under a humidified atmosphere containing 5% CO_2_. Then, H9c2 cardiomyocytes were passaged at a 1:2 ratio when they reached 80% confluence. All experiments were performed using H9c2 cardiomyocytes between 15 to 20 passage numbers.

For hypoxia, cells were grown to 80-90% confluence and then cultured under hypoxic conditions (94% N_2_, 5% CO_2_, and 1% O_2_) for 24 h at 37°C (17).

### Cell transfection

PCR was used to amplify the complete coding fragment of CLEC3B. The fragment was connected into the pcDNA3.1 vector (Invitrogen, USA) to construct pcDNA3.1-CLEC3B (pc-CLEC3B). The empty pcDNA3.1 was transfected as a negative control (pc-NC). Small interfering RNAs (siRNAs) against CLEC3B (si-CLEC3B) (F: 5′-CAGUGUAGCUAUGUCUCCCAAGUCU-3′, R: 5′-GACUUGGGAGACAUAGCUACACUG-3′) and negative control siRNA (si-NC) (F: 5′-CAGCGAUGUAUCUCUAACCGGUUCU-3′, R: 5′-AGAACCGGUUAGAGAUACAUCGCUG-3′) were designed and synthesized by Invitrogen. Lipofectamine^®^ 2000 transfection reagent (Invitrogen) was used to perform cell transfection. H9c2 cardiomyocytes were seeded into 6-well plates, and transfection began when the cardiomyocytes reached 70-80% confluence.

For gene overexpression, pc-CLEC3B (2 μg/well) or pc-NC (2 μg/well) and transfection reagent (12 μL/well) was diluted in 150 μL of serum-free Opti-MEM (Gibco, USA) for 5 min respectively. For gene silencing, si-CLEC3B (75 pmol/well) or si-NC (75 pmol/well) and transfection reagent (7.5 μL/well) were diluted in 100 μL of serum-free Opti-MEM for 5 min, respectively. Then, the diluted transfection reagent and plasmid/siRNA were mixed and incubated at 37°C for 30 min. The transfection mixture was added to the cell culture medium and mixed. The cell culture medium was replaced with fresh DMEM containing 10% FBS after 6 h of transfection, and the culture was continued for 48 h.

H9c2 cardiomyocytes were divided into 6 groups: control group (no hypoxia), hypoxia group (the cardiomyocytes underwent hypoxia as described above), pc-CLEC3B transfected cardiomyocytes cultured under hypoxia (pc-CLEC3B+hypoxia) group, pc-NC transfected cardiomyocytes cultured under hypoxia (pc-NC+hypoxia) group, si-CLEC3B transfected cardiomyocytes cultured under hypoxia (si-CLEC3B+hypoxia) group, and si-NC transfected cardiomyocytes cultured under hypoxia (si-NC+hypoxia) group. In order to further investigate the potential mechanisms of the effects of the pc-CLEC3B in H9c2 cardiomyocytes, an additional experiment was performed in which pc-CLEC3B was transferred into H9c2 cardiomyocytes with or without LY294002, an inhibitor of PI3K/Akt, and cultured under hypoxia. Each experiment was performed in triplicate.

### Cell proliferation

Cell proliferation was measured using the CCK-8 assay. H9c2 cardiomyocytes (6,000/well) were seeded into 96-well plates for 24 h. Then, cells were incubated in 10% CCK-8 solution for 1 h, and absorbance values were measured at 450 nm using a microplate reader (Thermo Fisher Scientific, USA). Each experiment was performed in triplicate.

### Apoptosis assay

Annexin V-APC/PI (Abnova, KA3807) staining and flow cytometry were performed to detect apoptosis of H9c2 cardiomyocytes. H9c2 cardiomyocytes (1×10^6^ cells/well) were seeded into 6-well plates for 24 h and then transfected and hypoxic-treated as described above. H9c2 cardiomyocytes were digested using 1 mL of 0.25% trypsin (EDTA free) (Beyotime, C0205) for 2 min. Next, H9c2 cardiomyocytes were washed with PBS buffer solution and centrifuged at 150 *g* for 5 min at room temperature. Then, H9c2 cardiomyocytes were resuspended in 500 μL of 1× binding buffer (1×10^6^ cells/mL) and added to appropriate tubes (1×10^5^ cells /tube). Five microliters of Annexin V-APC and 5 μL of PI solution were added to each tube for 15 min at room temperature in the dark. Subsequently, the cells were analyzed using a FACSCalibur^TM^ Flow Cytometer flow cytometry (BD Biosciences, USA) within 1 h. The total percentage of apoptotic cells was defined as the sum of both early apoptosis (Annexin V-APC positive, PI negative) and late apoptosis (Annexin V-APC/PI positive). Each experiment was performed in triplicate.

### Quantitative real-time PCR assay

TRIzol (Invitrogen) was used to isolate total RNA from H9c2 cardiomyocytes. RevertAid First Strand cDNA Synthesis Kit (Thermo Scientific, USA) was used to perform the reverse transcription (RT) reaction and TB Green^®^ Premix Ex Taq™ II (TaKaRa, Japan) was used to perform the qRT-PCR assay. The RT-qPCR results were analyzed as the fold change (2^-ΔΔCt^) and normalized with the expression of β-actin. The sequences of primers were synthesized, as listed in Table 1 (Tsingke, China). Each experiment was performed in triplicate.

**Table 1:** Primer information.

Primer	Sequence (5′ to 3′)
CLEC3B	F: ACGCCGCAGTCTGAGCTAGAGAATGA R: CGCCTTCCGAAGCCATGTCGTTGAG
β-actin	F: GAAGATCAAGATCATTGCTCC R: TACTCCTGCTTGCTGATCCA

F: forward; R: reverse.

### Western blot analysis

H9c2 cardiomyocytes were lysed with RIPA buffer for 30 min and quantified with a BCA protein assay kit. Equal amounts of protein samples were separated by 12% SDS-PAGE gels and then transferred onto a polyvinylidene difluoride (PVDF) membrane (Millipore). After blocking with 5% skim milk for 1 h at room temperature, the membranes were incubated overnight at 4°C with primary antibodies cleaved-caspase 3 (ab2302, 1:1,000 dilution), PI3K (ab191606, 1:1,000 dilution), p-PI3K (ab182651, 1:1,000 dilution), Akt (ab81283, 1:5,000 dilution), p-Akt (ab81283, 1:1,000 dilution), CLEC3B (ab202134, 1:1,000 dilution), and β-actin (ab8226, 1:1,000 dilution). Goat anti-rabbit IgG H&L secondary antibody (ab 205719, 1:2,000 dilution) was incubated with membranes at room temperature for 1 h. Bands of protein were visualized using the ECL kit (WBULS0500). Relative protein expression was quantified using Image-ProPlus 6.0 software (Media Cybernetics, USA) and normalized with the expression of β-actin. Each experiment was performed in triplicate.

### Statistical analysis

The quantitative data of this study are reported as means±SD. Statistical analysis was evaluated by SPSS 22.0 (IBM, USA) using Student's *t*-test or one-way analysis of variance (ANOVA). P<0.05 was considered to indicate a statistically significant difference.

## Results

### CLEC3B was downregulated in H9c2 cardiomyocytes with hypoxia

**Figure 1:**
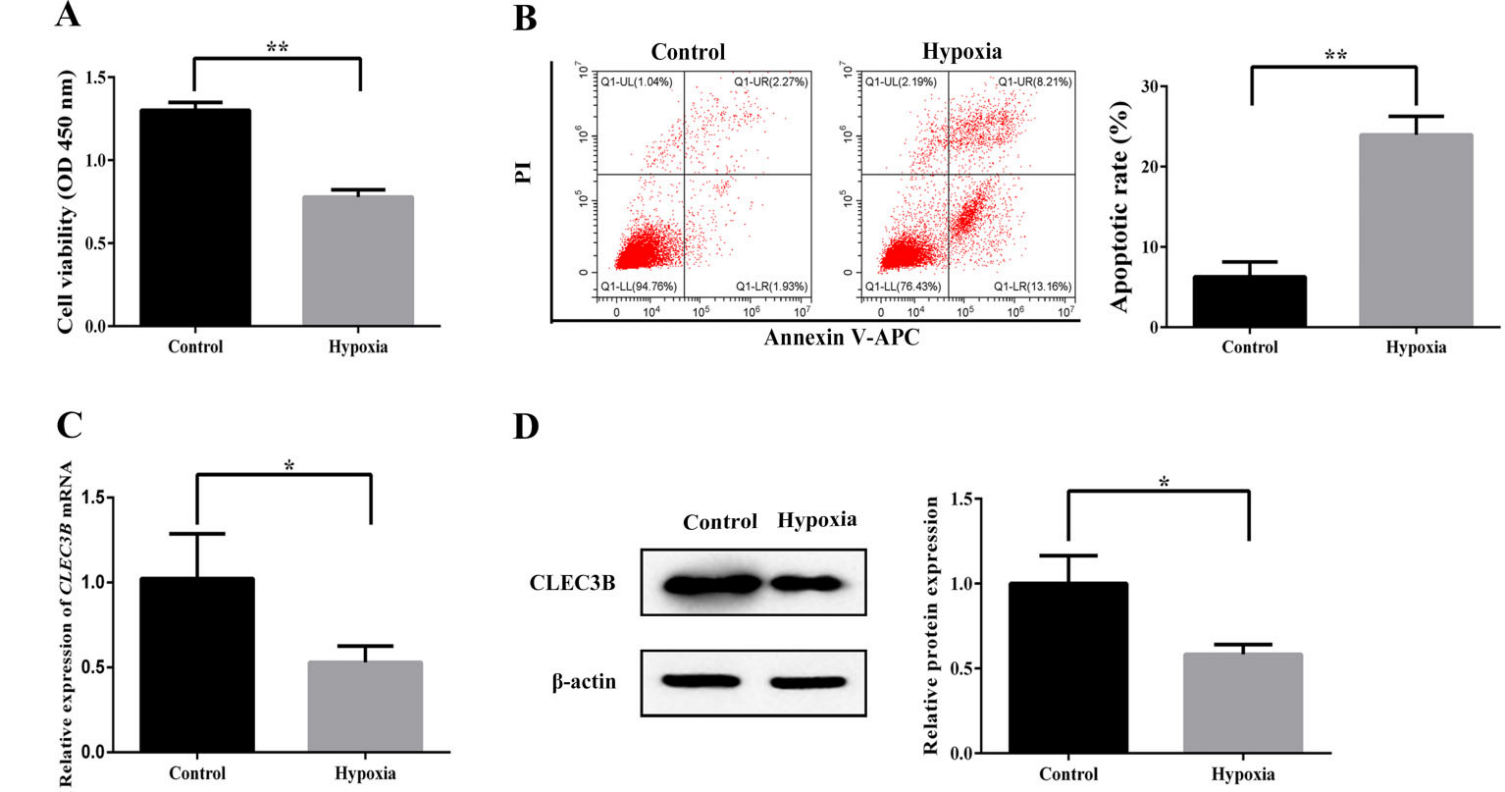
Effect of hypoxia on H9c2 cells. **A**, CCK8 assay was used to determine the effect of hypoxia on cell viability of H9c2 cells. **B**, Flow cytometry was used to evaluate the effect of hypoxia on apoptosis of H9c2 cells. **C**, QRT-PCR assay was performed to investigate the effect of hypoxia on *CLEC3B* mRNA expression in H9c2 cells. **D**, Western blot assay was used to detect the effect of hypoxia on the CLEC3B protein level in H9c2 cells. Data are reported as means±SD of 3 independent experiments. *P<0.05, **P<0.01 (Student’s *t*-test).

Cell viability of the hypoxia group was significantly decreased compared with the control group (P<0.01) (Figure 1A). There was a significant increase in apoptosis (Figure 1B) after 24 h of hypoxia compared with the cells cultured normally (P<0.01). The expression of CLEC3B was significantly decreased in the hypoxia group compared with the control group (P<0.05) (Figure 1C and D). These results indicated that the gene and protein expression levels of CLEC3B were decreased in H9c2 cardiomyocytes under hypoxia.

### Overexpression of CLEC3B promoted cell viability while loss of function of CLEC3B inhibited cell viability**in hypoxic H9c2 cardiomyocytes**

**Figure 2:**
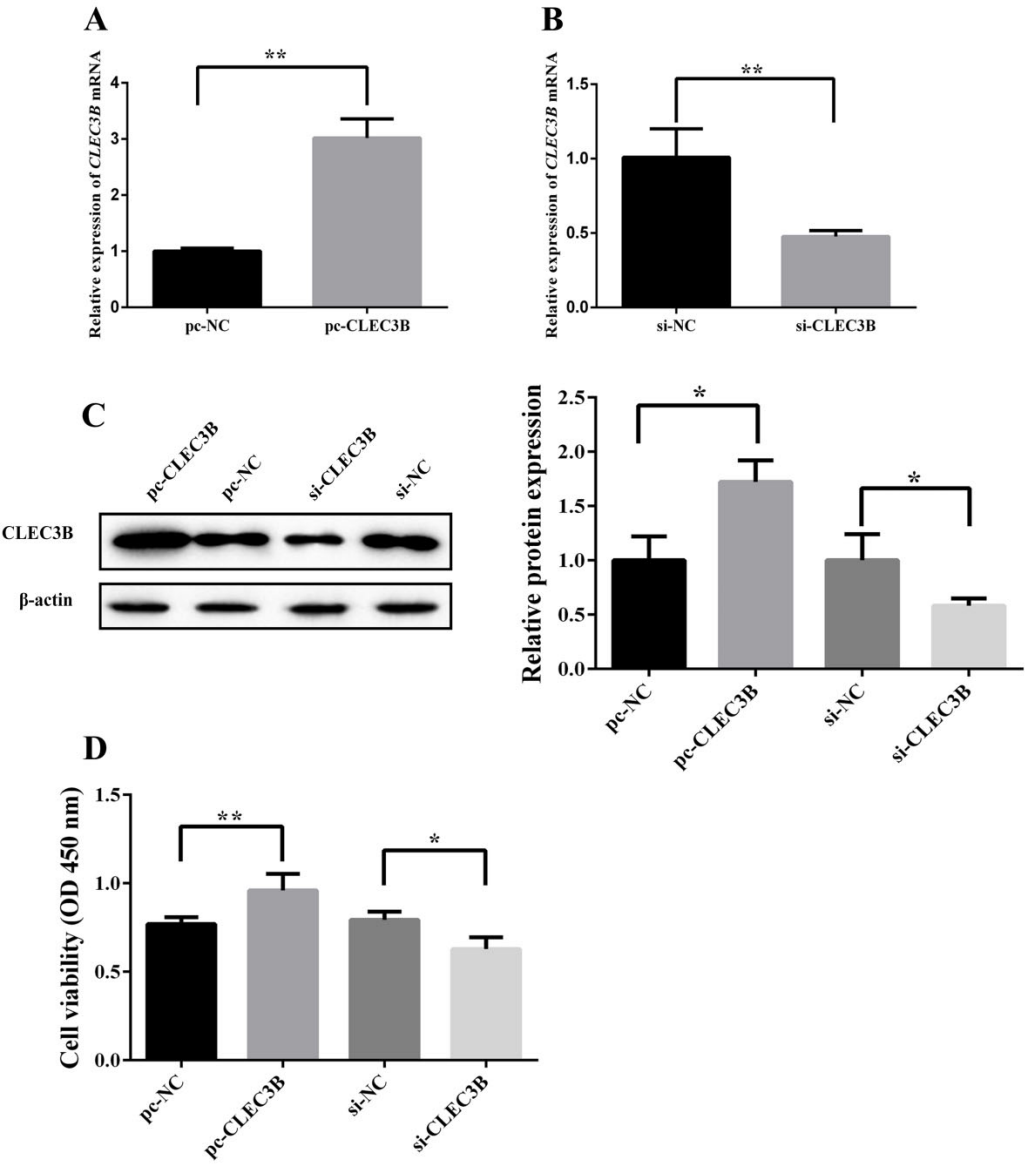
Effect of CLEC3B on cell viability of H9C2 cells with oxygen deficit. **A**, **B**, and **C**, Efficiencies of transfection of pc-CLEC3B and si-CLEC3B were verified by qRT-PCR and western blot assays. **D**, Cell viability of pc-NC (negative control), pc-CLEC3B, si-NC, or si-CLEC3B transfected H9c2 cells by CCK-8 assay. Data are reported as means±SD of 3 independent experiments. *P<0.05, **P<0.01 (Student’s *t*-test or ANOVA).

CLEC3B expression levels were significantly upregulated in the pc-CLEC3B group compared to the pc-NC group (Figure 2A and C). Meanwhile, the expression levels of CLEC3B were downregulated in the si-CLEC3B group compared with the si-NC group (Figure 2B and C). Moreover, CCK8 assay was used to determine the effect of CLEC3B on cell viability of H9c2 cardiomyocytes with oxygen deficit. As shown in Figure 2D, the cell viability of H9c2 cardiomyocytes was increased in the pc-CLEC3B group compared with the pc-NC group, while the cell viability of H9c2 cardiomyocytes was decreased in the si-CLEC3B group compared with the si-NC group. Our data demonstrated that transfection of pc-CLEC3B successfully increased cell viability of H9c2 cardiomyocytes with hypoxia while transfection of si-CLEC3B successfully decreased cell viability of H9c2 cardiomyocytes under hypoxia.

### Overexpression of CLEC3B inhibited apoptosis in hypoxic H9c2 cardiomyocytes

**Figure 3:**
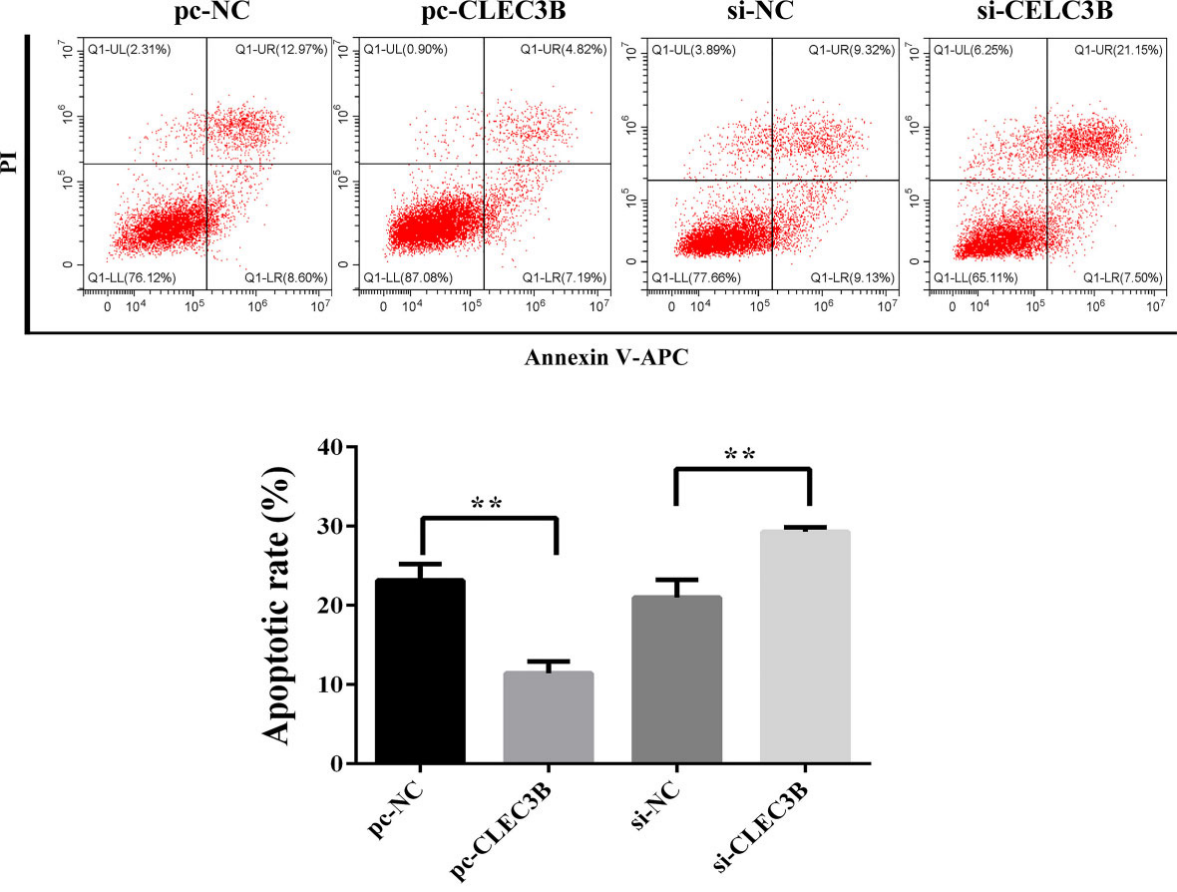
Effect of CLEC3B on apoptosis of H9c2 cardiomyocytes. Annexin V-APC/PI staining and flow cytometry were used to evaluate the effect of CLEC3B on apoptosis of H9c2 cardiomyocytes with hypoxia. Data are reported as means±SD of 3 independent experiments. **P<0.01 (ANOVA).

Apoptosis of H9c2 cardiomyocytes was decreased in the pc-CLEC3B group compared with the pc-NC group (P<0.01). Compared with the si-NC group, apoptosis of H9c2 cardiomyocytes was increased in the si-CLEC3B group (P<0.01) (Figure 3).

### CLEC3B activated PI3K/Akt signaling pathway and decreased cleaved-caspase 3 protein expression in hypoxic H9c2 cardiomyocytes

**Figure 4:**
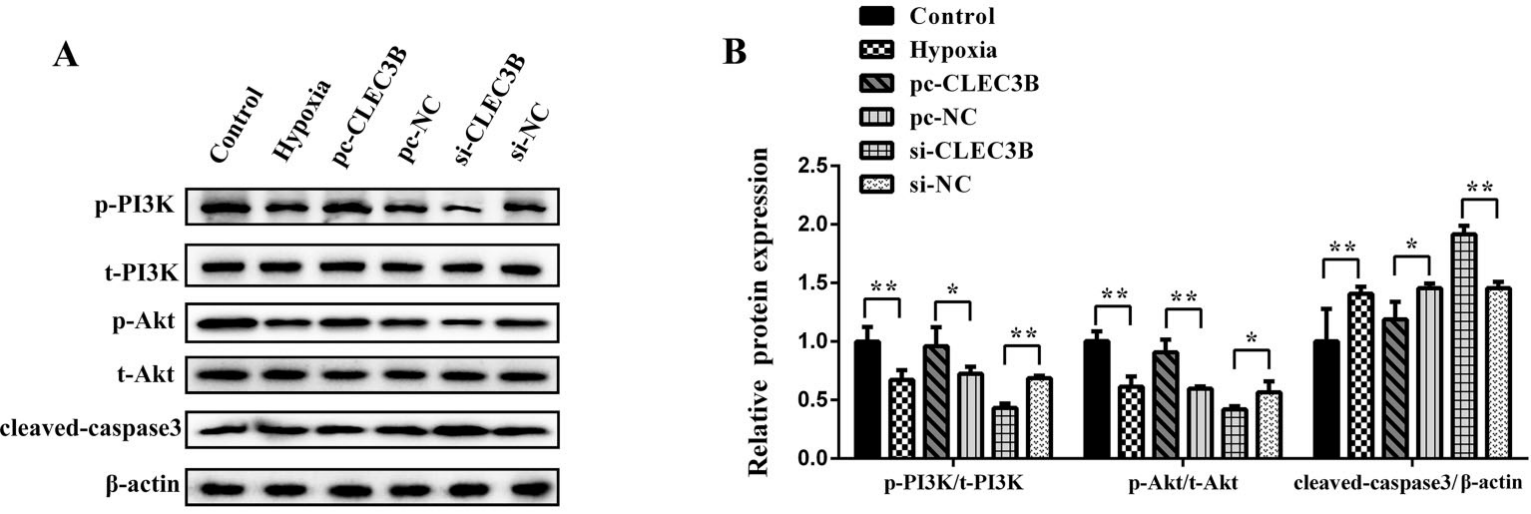
Effect of CLEC3B on the PI3K/Akt signaling pathway. **A**, Cells were transfected with pc-NC (negative control), pc-CLEC3B, si-NC, or si-CLEC3B and cultured in serum-free medium with hypoxia. Protein expression was evaluated by western blot analysis. **B**, Activation of the PI3K/Akt pathway was shown as the relative intensity of phosphorylated proteins/total proteins, and the relative intensity of cleaved-caspase 3 was normalized by β-actin. Data are reported as means±SD of 3 independent experiments. *P<0.05, **P<0.01 (ANOVA).

Among the H9c2 cardiomyocytes from the hypoxia group, the levels of p-PI3K/PI3K and p-Akt/Akt were markedly decreased compared with the control group (P<0.01), while the expression of cleaved-caspase 3 was increased compared with the control group (P<0.01). Levels of p-PI3K/PI3K and p-Akt/Akt were significantly increased (P<0.05 or P<0.01) while the expression level of cleaved-caspase 3 was decreased (P<0.05) by CLEC3B overexpression compared with the pc-NC group. At the same time, compared with the si-NC group, knockdown of CLEC3B decreased the levels of p-PI3K/PI3K and p-Akt/Akt (P<0.01 or P<0.05) and upregulated the expression of cleaved-caspase 3 (P<0.01) (Figure 4A and B). These data indicated that the effects of CLEC3B on hypoxia of H9c2 cardiomyocytes could be connected with the activation of the PI3K/Akt signaling pathway and the inhibition of cleaved-caspase 3, an apoptosis-related protein.

### Inhibition of PI3K/Akt signaling pathway reversed the anti-apoptotic influence of CLEC3B

**Figure 5:**
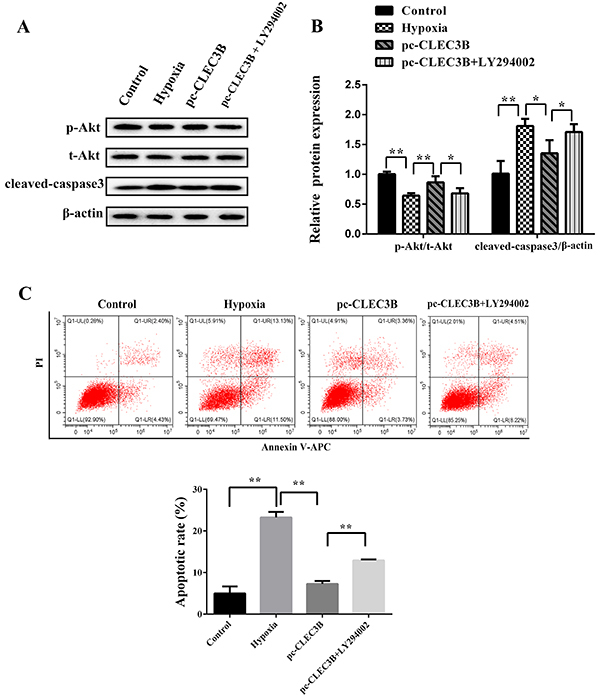
Effect of the inhibition of the PI3K/Akt signaling pathway on apoptosis of H9C2 cells. **A** and **B**, Western blot was performed to evaluate the effect of LY294002 on the PI3K/Akt signaling pathway. **C**, Annexin V-APC/PI staining and flow cytometry were used to evaluate the effect of the inhibition of the PI3K/Akt signaling pathway on apoptosis of H9c2 cells. Data are reported as means±SD of 3 independent experiments. *P<0.05, **P<0.01 (ANOVA).

The level of p-Akt/Akt was decreased while the expression of cleaved-caspase 3 was increased upon treatment with LY294002 compared with the pc-CLEC3B group (P<0.05) (Figure 5A and B). The apoptosis rate was significantly increased in the pc-CLEC3B + LY294002 group compared with the pc-CLEC3B group (P<0.01) (Figure 5C).

## Discussion

IHD is still one of the most dangerous diseases causing human death, and finding a therapeutic target for IHD is crucial (18,19). To investigate the molecular mechanisms of CLEC3B in IHD, H9c2 cardiomyocytes were used to simulate myocardial ischemia (20). Many factors affect the development of IHD (20,21). CLEC3B encodes tetranectin, which binds to plasminogen in a lysine-dependent manner and promotes the activation of plasminogen to regulate proteolytic processes (11,22). Here, we focused on CLEC3B and explored the role of CLEC3B in H9c2 cardiomyocytes under hypoxic conditions. Our results indicated that CLEC3B alleviated the injury of hypoxic H9c2 cardiomyocytes via the PI3K/Akt pathway.

Several studies reported that CLEC3B promotes myogenesis and inhibits cancer cell proliferation (11,23). In addition, the serum level of CLEC3B was downregulated in patients with coronary artery disease (16). Meanwhile, the expression level of CLEC3B was increased in hypoxic myocardial cells treated by miR-19a/19b mimics, two protective miRNAs of myocardial infarction (24). In this study, the protein and mRNA expression of CLEC3B were decreased in hypoxic H9c2 cardiomyocytes. Our data indicated, for the first time, that overexpression of CLEC3B increased cell viability and decreased apoptosis in hypoxic H9c2 cardiomyocytes.

It is well accepted that the PI3K/Akt pathway regulates cell proliferation, apoptosis, and metabolism (25). Liu et al. (26) discovered that isoflurane alleviates oxygen-glucose deprivation-induced H9c2 cell injury via activation of the PI3K/Akt pathway. Zhang et al. (19) found that emodin protects H9c2 cardiomyocytes from hypoxic injury by upregulating the expression of miR-138, which targeted mixed lineage kinase 3 (MLK3) and increased p-Akt expression. Therefore, the PI3K/Akt pathway might be a potential target for the treatment of myocardial ischemia. Moreover, cleaved-caspase 3 is an apoptosis-activating protein, which is regulated by the PI3K/Akt pathway and serves as a marker for the apoptosis-promoting effect (27,28). Therefore, we conjectured that the effect of CLEC3B on the proliferation and apoptosis of hypoxic H9c2 cardiomyocytes might be connected with the PI3K/Akt pathway and the expression of cleaved-caspase 3. Our data further demonstrated that the PI3K/Akt pathway was inhibited, and cleaved-caspase 3 expression was increased by oxygen deprivation in H9c2 cardiomyocytes. Overexpression of CLEC3B activated the PI3K/Akt pathway and reduced the expression of cleaved-caspase 3 while the silencing of CLEC3B caused the opposite results. Inhibition of the PI3K/Akt pathway reversed the protective effect of CLEC3B on hypoxic H9c2 cardiomyocytes.

In conclusion, this study indicated that CLEC3B was decreased in hypoxic H9c2 cardiomyocytes and it protected hypoxic H9c2 cardiomyocytes from apoptosis via the PI3K/Akt pathway. We provided the first connection between CLEC3B and the PI3K/Akt pathway. These results help to clarify potential treatment targets for IHD. Further animal and clinical studies should be performed in the future to validate this mechanism.
